# Plates Versus Bridges

**Published:** 1912-03-15

**Authors:** S. S. Doran


### PLATES VERSUS BRIDGES.

BY S. S. DORAN, L.D.S.

I trust you will forgive my temerity in taking the side of the plates in this discussion,but my remarks will consist of an attack on the present-day system of fixed bridge work from a hygienic standpoint. I feel nervous, somewhat like the man who is about to enter a lion's den for a wager. When he made the wager he felt brave, but as the time drew near he wished he had let some other man do the deed. So one feels when one is about to have a tilt at bridge work, the "bogey" the "Ideal," the ambition of every budding and painstaking dentist to be known to the public and his confreres as a bridge worker in contra-distinction to the ordinary practitioner who makes plates, but not so much money. Now, gentlemen:

You may remember that Dr. Hunter, pathologist, of Charing Cross, read a paper at Montreal on the Role of Sepsis and Antisepsis in medicine, in which he is said to have condemned crowns and bridge work in general as factors in oral sepsis. His paper has roused a great deal of discussion both in this country, and in America, but especially in the latter, as it was the so-called American dentistry that he attacked. In the November number of *Items of Interest* there is an editorial article in which the writer states that the editors of dental journals have been inundated with letters on the subject, and asking to take up the attack and slay the rash Briton, but the editor says—is there any truth in Dr. Hunter's paper? After quoting some papers by American dentists, *viz.*, Weston Price, 1901, D. M. L. Rhein, 1908, Prof. Bromwell, 1909, in which they all showed various failures in the different forms of bridge work, he cites the following:—In October, 1910, Dr. Herman Chayes published an article entitled "Experiences in bridge work," in connection with which he published illustrations made from photographs of sixteen pieces of bridge work. Of this class of work he says: "At best one is brought face to face with results that are unsatisfactory, incomplete, unwholesome and unclean," etc., etc. Of the specimens from which the illustrations are made he says: "These specimens are not the work of all-round incompetents; authors, clinicians, professors of prosthodontia figure among those at whose doors the existence of these specimens may be laid." Here then, we find not a denunciation of American dentistry by an English physician, but an arraignment of American dental incompetency by four American dentists. The writer winds up his article by saying that thousands of dentists do scientific, thoroughly aseptic and entirely sanitary work, and that thousands do the other thing and that perhaps the great underlying reason is that neither the surgeon, the physician, the dentist nor the patients themselves fully realize the dangers that threaten the whole body if the oral cavity be a secret storage place for oral sepsis. Mr. Badcock in the *B. D. Journal*, after stating that he is quite unable to properly fix a band for a collar crown under the gum margin, says, *re*fixed bridge work, that the only kind of bridge which can be considered perfectly clean is one which allows a free space between it and the gum it bridges over, bounded by rounded angles and convex surfaces which can be polished by means of a tape passed underneath it and drawn to and fro. With this statement I am in accordance; such a bridge is illustrated in Goslee's work, and is the only form of bridge that I consider ought to be inserted if we are to take oral sepsis into account; but even this bridge is not easy to keep clean, and although it is quite simple to clean from the dentist's point of view, or by the dentist, it is not so easy for the patient to pass tape through, as this structure is always at the back of the mouth. Those of you who wear bridges will appreciate this point. I purpose now to briefly run over a few statements in Goslee's book on bridge work.

Among the advantages of bridge work he gives (1) the removal of the many deficiencies associated with the substitution of missing natural teeth by means of the more common forms of plates which depend entirely upon contact for retention. The avoidance of mechanical abrasion of remaining teeth from clasps, or contact of plate, the preservation of their integrity and (mark well) the normal condition of the contiguous tissues. The more perfect reproduction of masticating surfaces, the removal of the impediment to speech, taste, &c., usually caused by plates. These he gives as *advantages*—we all know that patients get over the last-named quickly. Now listen to the disadvantages.

1. The possible necessity for devitalization of the pulp, mutilation of perhaps sound teeth.

The unnatural condition established by the secure fixation of the roots of two or more teeth. The additional stress to which the abutments are necessarily subjected.

The progressive degeneration of the peri-dental membrane which may ensue, causing looseness and resulting in subsequent loss of the teeth. The unhygienic and consequently unhealthy condition which may be endured by the wearing particularly of extensive fixed bridges.

In the chapter on hygienic considerations he states that all shoulders and pockets which would invite the accumulation of food products must be avoided, *all parts in contact* must be well adapted to the contiguous soft tissues. Lingual surfaces which will be sufficiently accessible to the bristles of a tooth brush to admit of its being kept *reasonably* (note the word) clean must be provided, and as large interproximal spaces as is consistent should exist. Now, gentlemen, you have heard the conditions that should exist, and I hope to show you that, save in one case, not one of these hygienic conditions is possible in any form of bridge work illustrated in this book or in either of the others mentioned.

(Mr. Doran then proceeded to show several illustrations taken from the text-book, which he claim substantiated his statement.)

Continuing, he said: One word on a pet aversion, the saddle bridge, defined by Goslee as that type wherein the body of the bridge which supports the dummies is conformed to the outline of, and placed in direct contact with, the contiguous soft tissue. He further states that the utility, when judiciously employed, is unquestionable.

In another chapter on the hygienic viewpoint of the saddle bridge he states—Contrary to the generally accepted belief that a saddle is decidedly unhygienic, such a device is frequently indicated in order to obtain a closer approach to hygienic results. Upon the removal of such bridges worn from 3 to 5 years—where the adaptation had been good, the surfaces of the saddles have been found clean and *comparatively* free from accumulation, except some little exfoliated epithelium. The patients had experienced no particularly (mark the word) unpleasant taste or offensive odours, and the tissues while presenting a slightly reddened, somewhat congested appearance, due, perhaps, to a superficial capillary stasis, as a result of pressure, indicated no *marked* evidences of soreness, inflammation, hypertrophy or resorption. Such results, however, could only be expected where a good close adaptation without irritating influences existed. One minute's reflection on this chapter will be enlightening. 1. Why were the bridges removed in three to five years? 2. How can a surface *be clean* and be only comparatively free from accumulations. It does not follow that because the patient experienced no offensive odours that his or her confidential friends did not.

There should be no question of an unpleasant taste, but as the author states that there was an accumulation and some exfoliated epithelium, one is forced to the conclusion that the latter must, in these particular instances, have given off an odour something like eau-de-cologne. Exfoliated epithelium means dead epithelial cells, consequent sepsis and attendant evils. I may mention that during the last year I have seen four saddle bridges—one made by a teacher of prosthetic dentistry and well made from a mechanical point of view, but the patient had a taste, a smell and an abscess due to accumulation, and this he was anxious to get rid of. Of the three other cases—one was badly made and filthy. The other two are still in the mouth, and as far as I know the patient is alive.

In conclusion, may I hope that these notes will stimulate a discussion from which we may all derive some benefit, but may I ask you not to discuss the bridge you have in your mind's eye, as it leaves the work-room, but rather the one that has been in the mouth some time or even the one that is hurried back to the work-room and to the acid pot!—*Dental Record.*

